# Wireless Communication Test on 868 MHz and 2.4 GHz from inside the 18650 Li-Ion Enclosed Metal Shell

**DOI:** 10.3390/s22051966

**Published:** 2022-03-02

**Authors:** Vlad Marsic, Tazdin Amietszajew, Petar Igic, Soroush Faramehr, Joe Fleming

**Affiliations:** Centre for Advanced Low-Carbon Propulsion Systems, Institute for Clean Growth and Future Mobility, Coventry University, Coventry CV1 5FB, UK; vlad.marsic@coventry.ac.uk (V.M.); taz.amietszajew@coventry.ac.uk (T.A.); petar.igic@coventry.ac.uk (P.I.); soroush.faramehr@coventry.ac.uk (S.F.)

**Keywords:** power line communication, 868 MHz, 2.4 GHz, Li-ion, battery, smart cell, wireless

## Abstract

As the RF communication on 18650 Li-ion cell level has not been reported due to its challenges and constrains, in this work, a valid wireless data link is demonstrated in an enclosed empty metal shell at 868 MHz and 2.4 GHz based on the IEEE 802.15.4 standard. The experimental tests are carried out using two generic unturned radiative structures, a wire loop fitted inside a cell shell, and an open terminal sub miniature version A (SMA), subsequently oriented vertically and horizontally relative to the ground plane. Based on signal strength indicator, bit error rate, and packet error rate, the test characterized a payload of 120 bytes at the highest speed of 150 kbps and 250 kbps supported by the IEEE 802.15.4 for the two communication frequencies. A MATLAB simulation is used in parallel to determine the three-dimensional radiative pattern of the two structures, whereas a three-ray model for multipath range propagation is implemented to complete the empirical experiments. It was demonstrated through testing communication of up to 10 m for both operating frequencies, proving the concept of wireless cell communication within short ranges, an essential feature for monitoring the health of each cell inside future electric vehicles (EVs).

## 1. Introduction

### 1.1. Health Monitoring in Energy Storage

**Figure 1:**
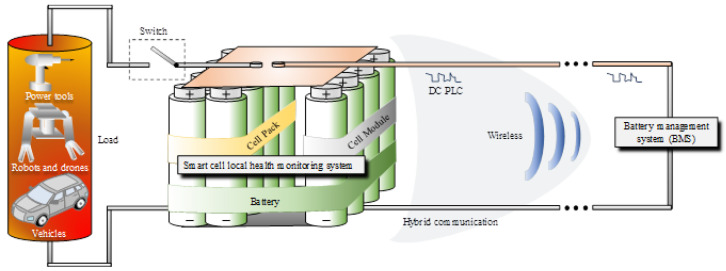
Battery monitoring system (BMS) research objectives.

Several factors account for the success of Li-ion rechargeable batteries, including the rise in demand for electric mobility (e-mobility) and grid energy storage for alternative power sources. Although use of the battery is an environmentally friendly and safe strategy, abusing the technology to the extreme usage may pose a health risk due to possible exposure to its hazardous chemical substances. Therefore, increasing efforts are made to control and monitor the technology by various implementations of Battery Management Systems (BMSs). With emphasis on reducing the overall weight and complexity in e-mobility, BMS technology adopts power line communication (PLC) [1,2,3], wireless monitoring [4,5,6,7] and hybrid techniques combining both methods [8]. Figure 1 illustrates the current research focus on BMS communication related to the rechargeable battery sub-hierarchical levels and the potential connected e-mobility representative loads.

BMS communication goes in hand with the growing interest in embedding sensors and in-situ cell monitoring. These range from various standard measurements such as voltage, current and temperature [9] to the mapping of light levels through advanced optical chromatic sensors [10,11] for preventing thermal runaway [12,13] or for battery state-of-health (SOH) diagnosis [14,15,16]. One of the major advantages of low-level monitoring at the cell level is the state-of-the-art demonstrated possibility of faster charging for current Li-ion batteries [17,18] or possible access to thermal modulation required for new fast-charging cells [19].

The wireless battery monitoring as a small network has been successfully demonstrated for 900 MHz [20] or 2.4 GHz [21,22] placing the RF transceiver outside the battery cell’s structure. However, wireless transmission from inside a small factor cell’s metal enclosure such as the ones encountered in 18650 (18 mm diameter), 21,700 (21 mm diameter) and 26,650 (26 mm diameter) models, requires more preliminary testing steps than for the plastic enclosures used in pouch and lead–acid batteries. A recent study demonstrates a wireless transmission data link by embedding inside a 21,700 cell the sensors and transceiver circuitry illustrating the potential and advantages of remotely reading data during cell’s cycling tests [23]. To the best of the author’s knowledge, a study investigating a possible wireless range for building a link budget that leads towards future network implementation has not been found despite acknowledging the usefulness of such a remote management approach. Moreover, due to its constrained space on millimetre scale between the cell’s top lid and sandwiched electrode, the antenna placed inside the cell is coupling with the metal enclosure. As all embedded RF circuits transmitting on the microwave spectrum and below will experience the same phenomenon, it will be also of interest to examine the radiation pattern by means of electromagnetic simulation since it has not been examined in prior studies.

### 1.2. Contribution and Paper Organization

In light of previous research that demonstrated various wireless data links [24,25] utilizing platforms external situated on different battery’s architecture level such as cells, packs, modules and batteries [5,6,20,25,26,27,28], this study will investigate the possibility of establishing a viable data link by transmitting from within an 18650-cell shell. Additionally, since examples of RF transceivers being used within a restrictive metal enclosure have been reported in both the communication [29,30] and radio location [31] domains, this study’s scenario proposes a challenging but realistic perspective. In this experiment, the selected frequencies of 868 MHz and 2.4 GHz will be tested on a range of 1 m up to 10 m using two types of radiative structures inside an 18650-cell shell: sub miniature version A (SMA) connector and a wire loop. Furthermore, the test will be conducted at close range of 0.1 m–0.2 m of a reflective surface emulating a mobile platform’s battery environment, with the radiative cell oriented vertically and then horizontally in relation to the surface. The tri-dimensional (3D) radiation pattern from insertion of antenna inside the cell’s shell will be visualized using a MATLAB electromagnetic simulation, whereas the experimental test results will be validated using RF modelling.

**Figure 2:**
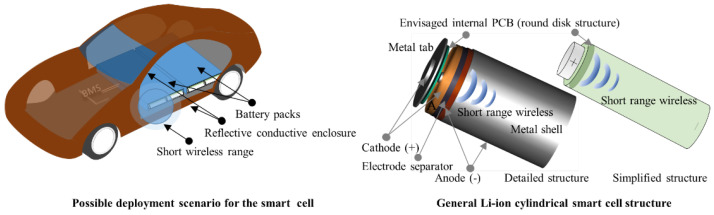
Battery monitoring system (BMS) at cell’s level implementation derived research objectives.

Towards the development of a network communication demonstrator based on smart cells, preliminary studies such as this one are essential, where simplifications and system-specific lab tests required lead to more advanced technological readiness levels (TRL) [32,33]. In Figure 2, we highlight briefly the particular case of a smart cylindrical Li-ion cell to be developed in the next TLR stage once we have proven that wireless communication can be successfully implemented inside a metal enclosed shell. The work’s metrics such as transmission range, frequency, data rate, payload derived from the received signal strength indicator (RSSI), bit error rate (BER) and packet error rate (PER), provides possible fundamental references for studies addressing similar structural functionality. In contrast, the constructed legacy will support and reinforce future laborious development efforts, such as PCB design, electrolyte-resisting coating, encapsulation inside a cell, and testing within a compact network for accessibility and reliability.

The work presented in this article is organised in four main sections. The introduction explains the study’s background and motivation. The second section presents the methodology, instruments, systems interconnectivity and characteristics. In this methodology section, the RF experimental ranging setup is accompanied by a MATLAB simulation which provides complementary data regarding the 3D resulted radiative elements and laboratory propagation profile based on a three-ray model. Based on simulations and empirical results, the third section discusses the results and the potential implications of data variation. The final section concludes with a summary of the study’s findings and a list of possible benefits.

## 2. Methodology

### 2.1. Experimental Setup

An experimental measurement of the spatial range will be carried out using a TI evaluation RF kit SMARTRF TRXEBK [30], which includes the wireless transceivers on 868 MHz CC1200 [34] and on 2.4 GHz CC2520 [35]. Both the transmitter (Tx) and receiver (Rx) ends will be controlled using a laptop running TI’s SmartRF Studio 7 [36]. By using a single RF kit for the two different operating frequencies, the results will be consistent on both hardware and software levels.

Since the purpose of our RF experiment is to evaluate the possibility of communication from within an 18650 Li-ion battery cell, we selected the conditions that are opposite of those that are most favourable to illustrate our hypothesis:

- An empty metal shell is constructed from nickel-plated cold-rolled steel, which is also used for the full Li-ion cell [37], including every element of its geometry.
- The radiative structures placed inside the cell’s metal shell, such as the SMA connector and the wire loop, are generic and not tuned or optimised for the specific selected frequencies of 868 MHz and 2.4 GHz.
- Using GFSK modulation on IEEE 802.15.4, a large payload of 120 bytes is transmitted with Tx power set to 0 dBm one hundred times at each test point, whereas employing the highest data rate supported by the utilised communication standard of 150 kbps for 868 MHz and 250 kbps for 2.4 GHz [38].

The non-optimal transmission test was designed so that if passed, under more optimised scenarios such as using detailed antenna and RF circuitry design and possible lower data rates and payloads, implementation will only need to support large network coexistence and data management.

Additionally, to prepare for the challenging environment that may be encountered when the cells are deployed inside a vehicle’s battery compartment, the wireless cell was tested vertically and horizontally at the laboratory’s concrete floor level. Under the SMA ends, a round, conductive flexible copper disk was positioned on top of a cardboard support to emulate the internal construction of such cells. The wire loop used alternatively with the SMA in the experiments presents a 9 mm radius and 0.3 mm thickness.

**Figure 3:**
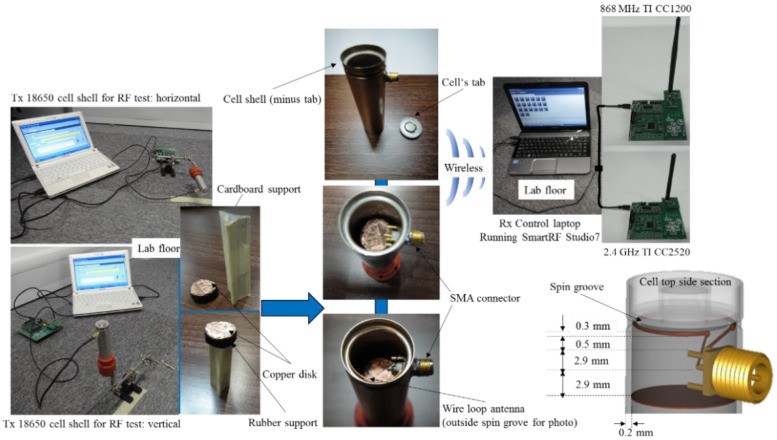
Experimental test systems used for 868 MHz and 2.4 GHz communication range testing inside the laboratory. The copper conductive surface/disk is fixed on a circular foam support which provides flexibility when inserted in the cell after previous SMA definitive positioning. Cardboard is used to raise the copper to the bottom of the SMA without touching it.

**Figure 4:**
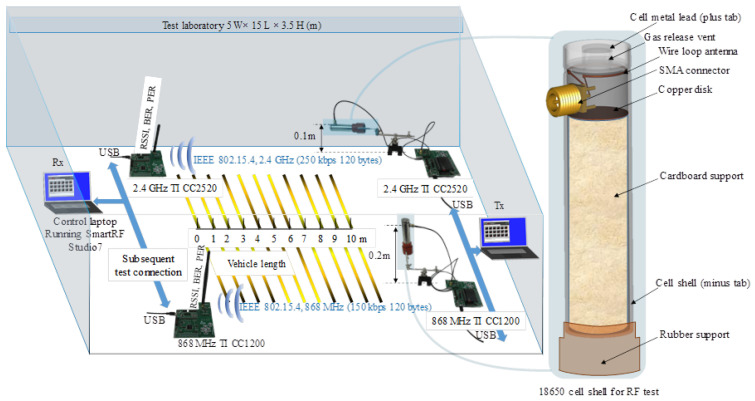
Experimental test setup for 868 MHz and 2.4 GHz communication range testing inside the laboratory. The radiative structure of an 18650 Li-ion shell vertical and horizontal oriented versus the ground plane is showed for the transmitter part (Tx), whereas the receiving unit (Rx) equipped with a standard antenna tuned for the communication frequencies is moved and interchanged subsequently accordingly.

An illustration of the used materials, structure and their dimensions is presented in Figure 3, whereas the experimental setup is shown in Figure 4, indicating the variable receiving points between 1 and 10 m, the subsequent connections to go through all the scenarios and the internal cell configuration used in the tests. In light of a potential electric vehicle transport scenario, the first 1 to 5 m range is of primary importance when analysing the empirical wireless range test. The mentioned range interval may prove useful internally for in-situ monitoring or for remote interrogation using proximity in cases where the process of recharging is based on separate infrastructure.

### 2.2. Simulation Setup

In this experiment, three-dimensional (3D) radiation pattern simulation is employed to determine the radiative characteristics of the SMA open ended and the wire loop when inserted inside a restricted reflective space, such as the 18650 metal shell. The simulation accounts for the effects of ground planes by including a large surface of 2 m by 2 m, whereas the reflections are maximised by considering the simulation materials as perfect electrical conductors (PECs). The focus of the simulation is to show the phenomena that may occur when employing similar radiative designs, however, since the radiative structures have not been specifically designed for the operating frequencies, their surface current distribution, impedances and voltage standing wave ratio (VSWR) are not key factors to be highlighted in testing.

**Figure 5:**
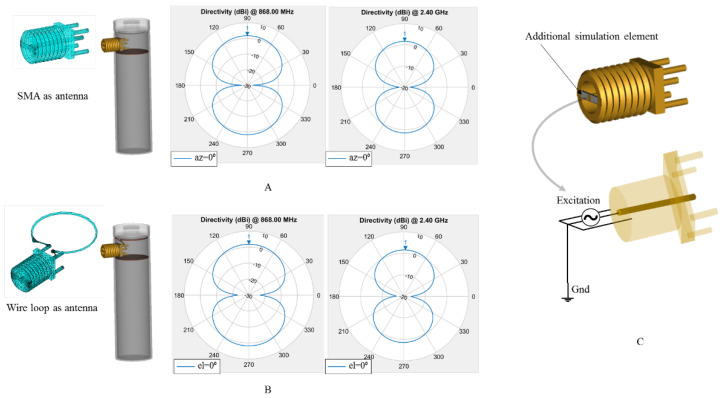
MATLAB RF test setup initial azimuth and elevation planes polar plots for 868 MHz and 2.4 GHz communication with the SMA as radiative element (**A**), the wire loop connected to the SMA (**B**) and the modification on the 3D design for the SMA to insert the simulation RF excitation source (**C**). Since the radiation pattern for dipole and loop antenna are resembling in shape with a torus while 90° offset from each other, the azimuth (az) plane for SMA is similar with the elevation (el) plane for the loop.

As a preliminary simulation measure before conducting the more complex 3D radiation pattern involving the cell structure and the reflective plane, the SMA connector and the wire loop attached to the SMA were implemented and tested separately before being attached to the cell’s shell structure, Figure 5. The azimuth and elevation planes in polar plots for both structures are illustrated in order to demonstrate that they act as described in classical RF literature. Three-dimensional representations of their attachment to the metal shell are shown separately in Figure 5A,B, whereas the additional connection to the SMA connector (i.e., closing the circuit between SMA’s central pin and external housing) to accommodate the RF simulation excitation point is shown in Figure 5C.

**Figure 6:**
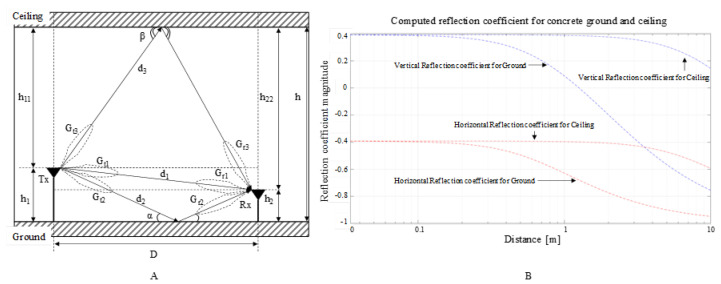
MATLAB signal attenuation simulation by using the Three-ray and Free space models for 868 MHz and 2.4 GHz for following the scenario described in (**A**), resulting in a computed reflection coefficient versus distance for the concrete ground and ceiling such illustrated in (**B**).

Radio line of sight (LOS) propagation can be numerically modelled by the free space path loss (FSPL) model [39]; however, it excludes the multipath effects. The secondary ground reflections at equal incident and reflective angles, including the FSPL model, can be modelled as shooting and bouncing rays (SBR) with the Two-ray model [40,41]. This model can then serve as a reference for derived multi-ray analysis, such as the Three-ray model [42], which accounts the ceiling reflection as well. Moreover, since the 3D ray tracing is subjective and highly sensitive to its shooting ray source model [43], Figure 6 shows the geometrically derived relationships [39] between the Two-ray model and the Three-ray implementation based on this study case:

(1) $$R_{ground}=\{\begin{array}{l} \frac{\varepsilon_{r}-\sqrt{\varepsilon_{r}-\cos^{2}\left(\alpha \right)}}{\varepsilon_{r}\sin\left(\alpha \right)+\sqrt{\varepsilon_{r}-\cos^{2}\left(\alpha \right)}},\;vertical\;polarisation\\ \frac{\sin\left(\alpha \right)-\sqrt{\varepsilon_{r}-\cos^{2}\left(\alpha \right)}}{\sin\left(\alpha \right)+\sqrt{\varepsilon_{r}-\cos^{2}\left(\alpha \right)}},\;horizontal\;polarisation \end{array};\;R_{ceiling}=\{\begin{array}{l} \frac{\varepsilon_{r}-\sqrt{\varepsilon_{r}-\cos^{2}\left(\beta \right)}}{\varepsilon_{r}\sin\left(\beta \right)+\sqrt{\varepsilon_{r}-\cos^{2}\left(\beta \right)}},\;vertical\;polarisation\\ \frac{\sin\left(\beta \right)-\sqrt{\varepsilon_{r}-\cos^{2}\left(\beta \right)}}{\sin\left(\beta \right)+\sqrt{\varepsilon_{r}-\cos^{2}\left(\beta \right)}},\;horizontal\;polarisation \end{array}$$

(2) $$d_{1}=\sqrt{{\left(h_{1}-h_{2}\right)}^{2}+D^{2}};\;d_{1}-d_{2}=\sqrt{{\left(h_{1}+h_{2}\right)}^{2}+D^{2}}-\sqrt{{\left(h_{1}-h_{2}\right)}^{2}+D^{2}};d_{1}-d_{3}=\sqrt{{\left(h_{11}-h_{22}\right)}^{2}+D^{2}}-\sqrt{{\left(h_{1}-h_{2}\right)}^{2}+D^{2}}$$

(3) $$\Delta \varphi_{1}=\frac{2\pi d_{1}}{\lambda };\;\Delta \varphi_{2}=\frac{2\pi (d_{1}-d_{2})}{\lambda };\Delta \varphi_{3}=\frac{2\pi (d_{1}-d_{3})}{\lambda }$$

(4) $$P_{r}=P_{t}{\left[\frac{\lambda }{4\pi }\right]}^{2}{\left|\frac{\sqrt{G_{t1}G_{r1}}e^{-j\Delta \varphi_{1}}}{d_{1}}+\frac{R_{ground}\sqrt{G_{t2}G_{r2}}e^{-j\Delta \varphi_{2}}}{d_{2}}+\frac{R_{ceiling}\sqrt{G_{t3}G_{r3}}e^{-j\Delta \varphi_{3}}}{d_{3}}\right|}^{2}$$

where *R* is the reflection coefficient for ground *R_ground_* and ceiling *R_ceiling_*, including the ray’s incidence associated angles *α* and *β*, *ε_r_* is the material relative electric permittivity, *d*_1*–*3_ is the ray path for each of the three casted rays (i.e., *r*1, *r*2 and *r*3), Δ*φ*_1*–*3_ is the phase difference derived from ray’s distance and wavelength λ, *P_r_* is the received power, *P_t_* is the transmit power, *G_t_*_1–3_ and *G_r_*_1–3_ are the Tx and Rx antenna, respectively, gains for the three casted rays (i.e., *r*1, *r*2 and *r*3).

When computing the numerical values of (1)–(4), the following assumptions have been made: as the ground plane and the ceiling for the measurement setup are made from concrete, *ε_r_* was set to the same value as suggested in [44]; using geometrical calculation methods in (2), ray paths can be predicted based on elevations of the Tx and Rx antennas from ground, such as 0.1–0.2 m, and room height *h* of 3.5 m, *h*_11_ *= h–h*_1_ and *h*_22_ *= h–h*_2_; considering the total separation distance between Tx and Rx is greater than the antenna heights, the signal phase difference ∆*φ* from (2) can be expressed as a truncation of Taylor’s series [34]; since the simulation serves as a reference for the experimental results, the transmitting and receiving gains *G_t_* and *G_r_* that are provided by the exact alignment of the radiation pattern with the incoming signal are all considered equal with one, whereas the transmitting power *P_t_* value is 1 mW.

In addition, the three terms of the sum in (4) can be identified as the free-space path loss (FSPL) added to the Two-ray flat ground reflection, with the final inclusion of the Three-ray flat ceiling reflection. The reflection coefficient computed and shown in Figure 6B provides an initial insight regarding the Three-ray simulation. The influence of the ground and ceiling are similar up to 1 m for both polarisations, whereas above that point the ceiling reflection dominates.

In this electromagnetic (EM) simulation, the MATLAB R2021b cross-disciplinary platform was selected due to its RF toolboxes providing the default tools for importing an external 3D computer-aided design (CAD) of an antenna and analysing it with various solvers depending on the electrical size of the scenario. As the overall scenario implemented through the radiative cell’s shell as an antenna on top of a large reflective platform of 2 m by 2 m delivered a mesh of more than 12,000 triangular faces, the method of moments (MoM) combined with physical optics (PO) was selected. The MoM-PO solver provided the best balance between simulation time i.e., approximatively 30 min each frequency and orientation scenario on a moderate computing platform equipped with a four-core processor Xeon E5-1620 V2 running at 3.7 GHz and 32 GB of RAM. Therefore, due to MATLAB platform selection, the 3D RF simulation enable the simulation run on an average personal computer (PC) configuration, reducing expenses incurred by the acquisition of dedicated RF software packages or custom hardware.

## 3. Results and Discussion

### 3.1. Empirical Experimental Results

**Figure 7:**
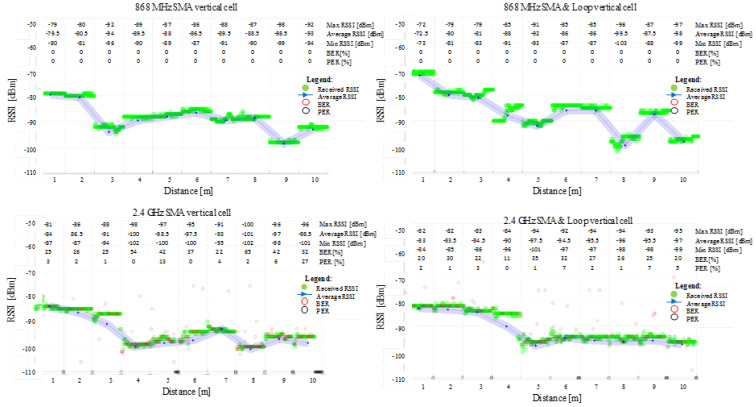
Experimental measurements received signal strength indicator (RSSI), bit error rate (BER) and packet error rate (PER) for 868 MHz and 2.4 GHz communication over the 1 m up to 10 m separation between the two RF transceivers. The radiative cell’s shell is in **vertical** orientation towards the ground plane.

**Figure 8:**
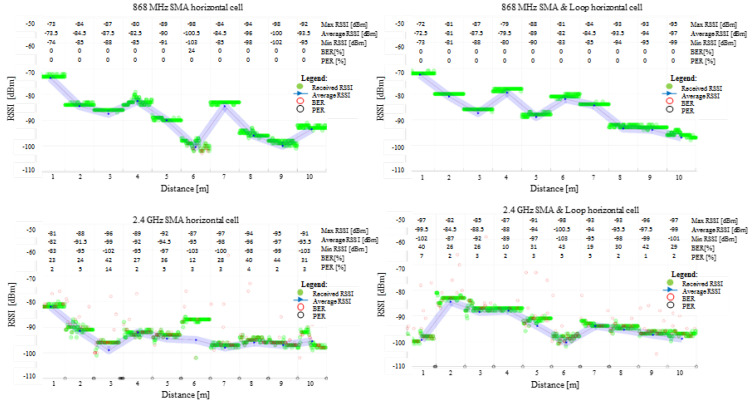
Experimental measurements received signal strength indicator (RSSI), bit error rate (BER) and packet error rate (PER) for 868 MHz and 2.4 GHz communication over the 1 m up to 10 m separation between the two RF transceivers. The radiative cell’s shell is in **horizontal** orientation towards the ground plane.

The results obtained for the field tests ranging from 1 m to 10 m for the cell’s shell’s radiative patterns at 868 MHz and 2.4 GHz, when the cells were vertically and horizontally oriented toward the reflective ground plane, are shown in Figure 7 and Figure 8. According to the graphs, the communication link behaviour indicates a better link at lower frequencies, supporting the theoretical simulation results. Due to its larger size and proximity to the top lid opening, the wire loop structure performs better than the open SMA terminal.

The results show no difference between the vertical and horizontal shell orientation of the cells within the interest range of 1 m to 5 m: in both frequency cases, the signal decreased close to the 3 m test point while displaying an ascending tendency to around −90 dBm for 868 MHz and −95 dBm for 2.4 GHz. Due to the fixed test points and integer metric system, small variations in signal amplitude at receiving locations can be attributed to the signal’s max and min relative to its spatial wavelength.

The 2.4 GHz communication encounters non-null BER and/or PER on all test ranges. The scenario, however, is not different from what might be corrected by simple acknowledgement and retransmission protocols. More than half of the 100 sent packages reached their destination intact, with the exception of the 4 m position on SMA in a vertical orientation, where the BER is slightly higher.

Although 868 MHz communication is a more suitable option for communication, it may be regulated through international standards at certain duty-cycle active times on certain channels [40,41], contrary to 2.4 GHz, which benefits from no on-air restrictions. In addition, since 868 MHz covers a wider range than 2.4 GHz with lower attenuation, it may require more resources at vehicle level for compliance with the current standards in terms of EMC/EMI. Accordingly, both tested operating frequencies are appropriate for battery cell level communication, although each one presents different advantages and challenges [45].

### 3.2. Simulation Results

**Figure 9:**
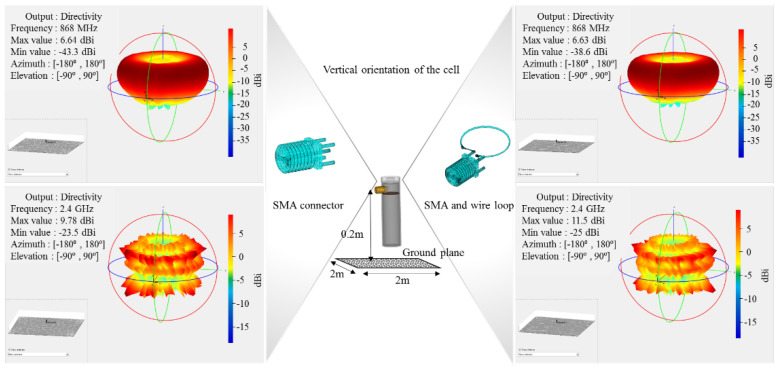
MATLAB 3D radiation pattern simulation for the cell’s shell radiative structure integrating the SMA and the wired loop for 868 MHz and 2.4 GHz over the ground plane in a **vertical** orientation.

**Figure 10:**
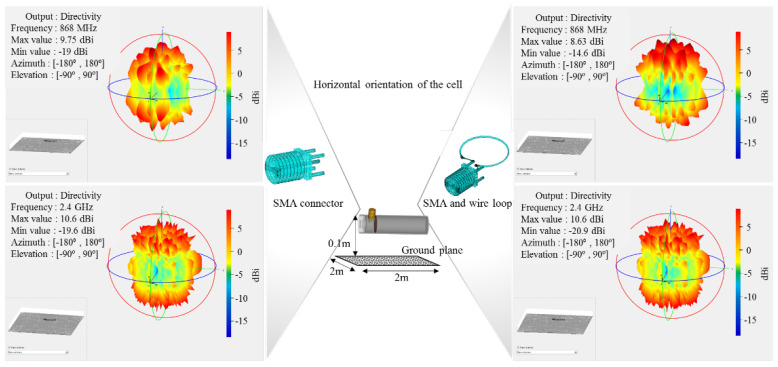
MATLAB 3D radiation pattern simulation for the cell’s shell radiative structure integrating the SMA and the wired loop for 868 MHz and 2.4 GHz over the ground plane in a **horizontal** orientation.

The results obtained for the 3D radiation pattern of the cell’s shell radiative structures at 868 MHz and 2.4 GHz at vertical and horizontal orientation towards the reflective ground plane are displayed in Figure 9 and Figure 10, respectively. One primary difference between the two communication frequencies is that 868 MHz exhibits fewer influences from the ground plane in both orientations, with a vertical polarisation pattern approaching a torus, whereas 2.4 GHz exhibits a furrow lobe profile. The proximity to the ground plane is reflected in the effects on the RX station as interference and short-range transmission detection.

With the cell orientated horizontally compared with the ground plane, there is a higher coupling with the plane than the vertical configuration, both communication frequencies undergoing furrow lobe profiles while their horizontal maxima is rotated 90 degrees from the precedent set. The presence of the highly irregular profile at Tx i.e., multiple lobes and nulls, will manifest at Rx by inducing shadowing effects when the receiving station’s orientation will align an area between the Tx lobes. As a consequence of the 3D radiative profile showing a more regular distribution of intensity in vertical orientation compared with horizontal, the first assumption is that vertical orientation will support better reception of signals. However, as confirmed by experimental and further three-ray propagation model, the signal variation is more dependent on the maxima-minima fields recorded for each simulation and the resulting lobe positions rather than the radiation profile uniformity or smoothness.

**Figure 11:**
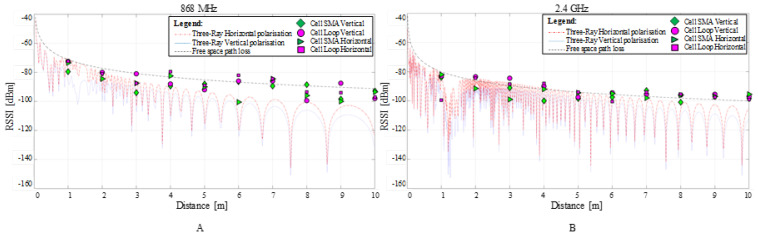
MATLAB signal attenuation simulation by using the Three-Ray and Free space models for 868 MHz (**A**), and 2.4 GHz (**B**), between the concrete ground.

Based on the Three-ray model for the two communication frequencies shown in Figure 11, the vertical and horizontal polarization simulated by the model are in agreement with other previous studies examining similar settings, such as experiments inside concrete tunnels [46] or buildings [47]. The FSPL line plot defines the simulation upper limit while the maxima and minima of the Three-ray model known as multipath fading define the lower bound. Since the 2.4 GHz frequency wavelength is approximatively 2.8 times smaller than for 868 MHz, the number of signal distortions due reflections is larger than for the latter case.

When plotting the experimental RSSI averaged values over the propagation simulation results, it can be observed that some values for both investigated frequencies exceed the FSPL line. For the above high values, since the simulation does not include any gain above units derived from antenna position alignment, the Tx and Rx positions should be assumed under optimised alignment. Although for the empirical measurements, the metric distance increased with a 1 m step did not account for the multipath fading, a possible Tx-Rx positioning optimisation can be achieved by following the Thee-ray pattern for which the spatial step is set to one tenth of the wavelength.

## 4. Conclusions and Further Work

In this study, we demonstrated through empirical experimentation that wireless communication can be accomplished down to the difficult level of the 18650-cell constrained-space metal enclosure while delivering useful ranges for e-mobility platforms. The two communication frequencies of 868 MHz and 2.4 GHz are shown to support data links up to 10 m, despite the fact that the radiative structures used to demonstrate these properties were not specifically adjusted for this application. This information is fundamental for link budget calculations for a compact smart cell network implementation and deployment. This work may also serve as a reference point for applications that use embedded antennas within similar restrictive metal enclosures.

By using the 3D MATLAB simulation, it was possible to illustrate how microwave antennas would be affected by small restrictive metal environments, such as the 18650-cell shell. Through simulation, it has been found that when radiative elements are packed together, they will couple, changing the initial individual radiative elements radiation pattern. Smart cells packed in a dense battery network may encounter the same phenomenon, resulting in a wireless-wired hybrid communication method based on both radiative and conductive propagation. The good agreement between the discrete experimental and the three-ray simulation values for the tested general radiative elements makes multi-ray models a promising choice for link budget calculations with applications in wireless battery management systems used in e-mobility.

As this study demonstrates an available range up to 10 m for a common low power 868 MHz or 2.4 GHz RF transceiver enclosed inside a smart cell potentially used in vehicular transportation, indirect usage of the technology for traffic monitoring, parking occupancy, car classification, etc., may be explored and exploited in future applications.

Research such as this is critical for future implementations of wireless BMS technology at the cell level, which will deliver the capability of fast charging and improved safety while providing additional indirect benefits through infrastructure sensing in the future smart cities.

## Data Availability

The datasets generated and analysed during this study are available from the corresponding author on a reasonable request, but restrictions apply to the commercially confident details.

## References

[1] Thomas F.L., Guenter S., Guenter H., Rose M., Jossen A. (2021). Power Line Communications for Automotive High Voltage Battery Systems: Channel Modeling and Coexistence Study with Battery Monitoring. Energies.

[2] Timothy A.V., Begum G., EH S.J., James M. (2021). Development of an in-vehicle power line communication network with in-situ instrumented smart cells. Transp. Eng..

[3] Koshkouei M.J., Kampert E., Moore A.D., Higgins M.D. (2021). Evaluation of an in situ QAM-based Power Line Communication system for lithium-ion batteries. IET Electr. Syst. Transp..

[4] Gozdur R., Przerywacz T., Bogdański D. (2021). Low Power Modular Battery Management System with a Wireless Communication Interface. Energies.

[5] Mattia R., Meng J., Gherman T., Grandi G., Teodorescu R. (2019). Smart battery pack for electric vehicles based on active balancing with wireless communication feedback. Energies.

[6] Huang L.T., Ha D.S., Cho H. Low Power Design of a Wireless Sensor Node to Monitor Electric Car Batteries. Proceedings of the IECON 2018—44th Annual Conference of the IEEE Industrial Electronics Society.

[7] Mathew S.A., Prakash R., John P.C. A smart wireless battery monitoring system for Electric Vehicles. Proceedings of the 2012 12th International Conference on Intelligent Systems Design and Applications (ISDA).

[8] Alapati S.V., Nadella I., Bobba P.B., Upadhayay M.D. (2019). Development of wireless charging system along with power line communication used in Electric Vehicles. E3S Web Conf..

[9] Lidiya K., Buchberger T., Diehl S., Ehrensberger M., Hanzl C., Hartmann C., Hölzle M., Kleiner J., Lewerenz M., Liebhart B. (2021). Critical review of intelligent battery systems: Challenges, implementation, and potential for electric vehicles. Energies.

[10] Fleming J., Amietszajew T., McTurk E., Towers D.P., Greenwood D., Bhagat R. (2018). Development and evaluation of in-situ instrumentation for cylindrical Li-ion cells using fibre optic sensors. HardwareX.

[11] Rente B., Fabian M., Vidakovic M., Liu X., Li X., Li K., Sun T., Grattan K.T. (2020). V Lithium-Ion battery state-of-charge estimator based on FBG-based strain sensor and employing machine learning. IEEE Sens. J..

[12] Fleming J., Amietszajew T., Charmet J., Roberts A.J., Greenwood D., Bhagat R. (2019). The design and impact of in-situ and operando thermal sensing for smart energy storage. J. Energy Storage.

[13] Jichao H., Wang Z., Ma F., Yang J., Xu X., Qu C., Zhang J., Shan T., Hou Y., Zhou Y. (2021). Thermal Runaway Prognosis of Battery Systems Using the Modified Multi-Scale Entropy in Real-World Electric Vehicles. IEEE Trans. Transp. Electrif..

[14] Amietszajew T., Fleming J., Roberts A.J., Widanage W.D., Greenwood D., Kok M.D.R., Pham M., Brett D.J.L., Shearing P.R., Bhagat R. (2019). Hybrid thermo-electrochemical In Situ instrumentation for lithium-ion energy storage. Batter. Supercaps.

[15] Wu Y., Wang Y., Yung W.K.C., Pecht M. (2019). Ultrasonic health monitoring of lithium-ion batteries. Electronics.

[16] Surya S., Rao V., Williamson S.S. (2021). Comprehensive Review on Smart Techniques for Estimation of State of Health for Battery Management System Application. Energies.

[17] Amietszajew T., McTurk E., Fleming J., Bhagat R. (2018). Understanding the limits of rapid charging using instrumented commercial 18650 high-energy Li-ion cells. Electrochim. Acta.

[18] Mohammed A.-S., Bartosz P., Maciej Z., Karwat A., Pol M., Chełchowski Ł., Van Mierlo J., Berecibar M. (2021). Slow and Fast Charging Solutions for Li-Ion Batteries of Electric Heavy-Duty Vehicles with Fleet Management Strategies. Sustainability.

[19] Yang X.G., Liu T., Wang C.Y. (2021). Thermally modulated lithium iron phosphate batteries for mass-market electric vehicles. Nat. Energy.

[20] Lee M., Lee J., Lee I., Lee J., Chon A. Wireless battery management system. Proceedings of the World Electric Vehicle Symposium and Exhibition (EVS27).

[21] Rauniyar A., Irfan M., Saputra O.D., Kim J.W., Lee A.R., Jang J.M., Shin S.Y. (2017). Design and development of a Real-Time monitoring system for multiple lead–acid batteries based on Internet of things. Future Internet.

[22] Bacquet S., Maman M. Radio Frequency Communications for Smart Cells in Battery Pack for Electric Vehicle. Proceedings of the 2014 IEEE International Electric Vehicle Conference (IEVC).

[23] Yang L., Li N., Hu L., Wang S., Wang L., Zhou J., Song W.L., Sun L., Pan T.S., Chen H.S. (2021). Internal field study of 21700 battery based on long-life embedded wireless temperature sensor. Acta Mech. Sin. Xuebao.

[24] Samanta A., Williamson S.S. (2021). A Survey of Wireless Battery Management System: Topology, Emerging Trends, and Challenges. Electronics.

[25] Huang X., Acharya A.B., Meng J., Sui X., Stroe D.-I., Teodorescu R. Wireless Smart Battery Management System for Electric Vehicles. Proceedings of the IEEE Energy Conversion Congress and Exposition (ECCE).

[26] Kim H., Shin K.G. Efficient sensing matters a lot for large-scale batteries. Proceedings of the IEEE/ACM Second International Conference on Cyber-Physical Systems.

[27] Takeuchi T., Terada T. (2014). Evaluation of Wireless Communication Performance in a Li-ion Battery System. J. Auto Contr. Eng..

[28] Otto A., Rzepka S., Mager T., Michel B., Lanciotti C., Günther T., Kanoun O. (2012). Battery management network for fully electrical vehicles featuring smart systems at cell and pack level. Advanced Microsystems for Automotive Applications.

[29] Ibrahim M.A., Hassan G., Hassanein H.S., Obaia K. A wireless sensor platform for industrial non-hermetic metallic enclosures. Proceedings of the 13th International Wireless Communications and Mobile Computing Conference (IWCMC).

[30] Kumar P.S., Xie L., Soong B.-H., Lee M.Y. (2018). Feasibility for utilizing IEEE 802.15. 4 compliant radios inside rotating electrical machines for wireless condition monitoring applications. IEEE Sens. J..

[31] Vlad M., Erik K., Higgins M.D. Position Discrimination of a 2.4 GHz IEEE 802.15. 4 RF Mobile Source Inside-Outside a Vehicle. Proceedings of the IEEE International Conference on Smart Applications, Communications and Networking (SmartNets).

[32] Mankins J.C. (1995). Technology readiness levels. White Pap. April.

[33] Straub J. (2015). In search of technology readiness level (TRL) 10. Aerosp. Sci. Technol..

[34] Texas Instruments CC1200: Low Power and High Performance Wireless Transceiver. https://www.ti.com/product/CC1200.

[35] Texas Instruments CC2520: Second Generation 2.4 GHz ZigBee/IEEE 802.15.4 Wireless Transceiver. https://www.ti.com/product/CC2520.

[36] Texas Instruments SmartRF Studio 7 Documentation. http://software-dl.ti.com/lprf/smartrftm_studio/docs/help/html/srfstudio.html.

[37] Wang L., Yin S., Yu Z., Wang Y., Yu T.X., Zhao J., Xie Z., Li Y., Xu J. (2018). Unlocking the significant role of shell material for lithium-ion battery safety. Mater. Des..

[38] Ramonet A.G., Noguchi T. (2020). IEEE 802.15. 4 Now and Then: Evolution of the LR-WPAN Standard. ICACT Trans. Adv. Commun. Technol..

[39] Goldsmith A. (2005). Wireless Communications.

[40] Lu N.H. Linearized, unified two-ray formulation for propagation over a plane earth. Proceedings of the Sensors for Industry Conference.

[41] Zöchmann E., Guan K., Rupp M. Two-ray models in mmWave communications. Proceedings of the IEEE 18th International Workshop on Signal Processing Advances in Wireless Communications (SPAWC).

[42] Saleh F. A three ray propagation model for line of sight PCS and micro-cellular services. Proceedings of the 6th International Symposium on Personal, Indoor and Mobile Radio Communications.

[43] Vlad M., Kampert E., Higgins M.D. Ray Tracing 3D Source Modelling for Optical Reflectance Sensing with Wireless Ranging Application. Proceedings of the IEEE International Symposium on Robotic and Sensors Environments (ROSE).

[44] ITU (2021). Effects of building materials and structures on radiowave propagation above about 100 MHz. Recommendation ITU-R P.2040-2.

[45] Velagapudi P., Eravatri B.C., Mantri M.B., Mani V.V. Performance analysis of various IEEE 802.15. 4 PHYs under Rayleigh fading channel. Proceedings of the International Conference on Advanced Computing and Communication Systems.

[46] Zhou C., Plass T., Jacksha R., Waynert J.A. (2015). RF Propagation in Mines and Tunnels: Extensive measurements for vertically, horizontally, and cross-polarized signals in mines and tunnels. IEEE Antennas Propag. Mag..

[47] Rudd K., Craid R., Ganley M., Hartless R. (2014). Building Materials and Propagation Final Report-Ofcom.

